# The Economic Burden of Road Traffic Injuries on Households in South Asia

**DOI:** 10.1371/journal.pone.0164362

**Published:** 2016-10-21

**Authors:** Khurshid Alam, Ajay Mahal

**Affiliations:** 1 Murdoch Childrens Research Institute, 50 Flemington Road, Parkville, Victoria, 3052, Australia; 2 Department of Pediatrics, The University of Melbourne, 50 Flemington Road, Parkville, Victoria, 3052, Australia; 3 Nossal Institute for Global Health, The University of Melbourne, 161 Barry Street, 4th Floor, Carlton, VIC 3053, Australia; 4 Department of Epidemiology and Preventive Medicine, Monash University, Level 6, The Alfred Centre, 99 Commercial Road, Melbourne, VIC 3004, Australia; RTI International, UNITED STATES

## Abstract

Globally, road traffic injuries accounted for about 1.36 million deaths in 2015 and are projected to become the fourth leading cause of disability-adjusted life years (DALYs) lost by 2030. One-fifth of these deaths occurred in South Asia where road traffic injuries are projected to increase by 144% by 2020. Despite this rapidly increasing disease burden there is limited evidence on the economic burden of road traffic injuries on households in South Asia. We applied a novel *coarsened exact matching* method to assess the household economic burden of road traffic injuries using nationally representative World Health Survey data from five South Asian countries- Bangladesh, India, Nepal, Pakistan and Sri Lanka collected during 2002–2003. We examined the impact of road traffic injuries on household out-of-pocket (OOP) health spending, household non-medical consumption expenditure and the employment status of the traffic injury-affected respondent. We *exactly* matched a household (after ‘coarsening’) where a respondent reported being involved in a road traffic injury to households where the respondent did not report a road traffic injury on each of multiple observed household characteristics. Our analysis found that road traffic injury-affected households had significantly higher levels of OOP health spending per member (I$0.75, *p*<0.01), higher OOP spending on drugs per member (I$0.30, *p* = 0.03), and higher OOP hospital spending per member (I$0.29, *p*<0.01) in the four weeks preceding the survey. Indicators of “catastrophic spending” were also significantly higher in road traffic injury-affected households: 6.45% (*p*<0.01) for a threshold of OOP health spending to total household spending ratio of 20%, and 7.40% (*p*<0.01) for a threshold of OOP health spending to household ‘capacity to pay’ ratio of 40%. However, no statistically significant effects were observed for household non-medical consumption expenditure, and employment status of the road traffic injury-affected individual. Our analysis points to the need for financial risk protection against the road traffic injury-related OOP health expenditure and a focus on prevention.

## Introduction

Road traffic injuries accounted for nearly 1.36 million deaths worldwide in 2015, and ranked as the eighth leading cause of years of life lost (YLLs) in that year [1]. Globally, the burden of road traffic injuries, measured in terms of number of disability-adjusted life years (DALYs) ranked ninth in 2015 but are projected to be the fourth leading cause of disease burden by 2030 [2, 3]. The low- and middle-income countries (LMICs) account for a disproportionate share of traffic injuries worldwide with nearly 85% of deaths and 90% of DALYs lost [4]. One-fifth of these deaths occurred in South Asia with road traffic injuries being the 11^th^ leading cause of DALYs lost in the region in 2010 [5, 6]. The reason for the rising death toll in LMICs such as in South Asia is rapid urbanization and motorization associated with rapid economic growth [7]. Existing projections suggest a 144% increase in road traffic deaths in South Asia over the period from 2000 to 2020, so we can expect the relative significance of road traffic injuries in South Asia’s disease burden to rise rapidly in the future [8]. Globally, more than 50% of road traffic injury-related deaths occur in the age group 15–49 years, an economically productive period [7, 9]. A similar age distribution has been noted in traffic injury deaths and related hospitalizations in countries of South Asia [9–12]. Road traffic injuries can also require expensive hospital-based treatment, including trauma care [13]. As a consequence, studies estimating the cost-of-illness of road traffic injuries (direct costs of treatment and productivity losses) or the value of a statistical life arrive at measures of aggregate economic impacts that are staggering, ranging from 1.3% to 3.0% of gross domestic product (GDP) annually in South Asia [14]. Estimates of national level economic impacts using the monetary value of a statistical life and cost-of-illness methods have faced a number of methodological challenges [15]. In addition, such estimates also cannot tell us about the impact of road traffic injuries on the economic burden faced by the affected households, because some of the burden of injury care may be borne by others (e.g., subsidized public facilities), or may be pushed into the future as households with injured members incur debt or sell assets to finance care, and household members may experience earnings losses [16].

There is a limited literature on the household level economic burden in LMICs including South Asian countries, but much of it focuses on the out-of-pocket (OOP) medical treatment expenses of households with severely injured and/or hospitalized patients for small population sub-groups. A recent cohort study among 155 trauma patients in a tertiary care hospital of northern India shows that high OOP expenditures pose major economic burden for the affected families [17]. A comparative study of traffic injury patients in Bangladesh and the district of Bangalore (India) found average household OOP expenses ranging from US$52 to US$93 in Bangladesh and US$380 to US$780 in Bangalore, respectively [12, 18]. In Hyderabad (India), a study of 723 patients in three hospitals showed that the median OOP medical expenditure per traffic injury case was US$169 [19]. A study of 95 traffic accident cases in Chandigarh (India) showed that OOP medical expenses averaged US$100 [20]. In Nepal, a study of 505 injury patients (40% of whom were traffic injury cases) reported OOP expenses per patient of US$45 in 2008 [11]. Razzak *et al*. (2011) found in their study of traffic injury patients at five trauma centers in Karachi (Pakistan) OOP healthcare costs of US$ 271 per patient [21]. Finally, a household survey-based study of road traffic injury cases in Kandy (Sri Lanka) found average OOP health expenses of US$300 [22].

Few studies have gone beyond the issue of OOP treatment expenses for road traffic injuries in South Asia. Two recent studies found that in more than one-half of the households with traffic injury cases, income and food consumption declined, and indebtedness increased [12, 14]. However, these households did not report specific amounts, merely ‘yes/no’ responses to survey questions. The studies did report the magnitude of the loss of work for the injured person due to injury (ranging from 89 days to 124 days in Bangladesh and 133 days to 180 days in Bangalore). Razzak *et al*. (2011) crudely estimated the value of work time lost by the injured person and their attendants to be $67 per injured patient in Karachi [21]. Nithershini *et al*. (2012) did so for injured persons in Kandy (Sri Lanka), by multiplying work-time lost with a salary indicator [22].

The above analyses of the economic impacts of traffic injuries in South Asia (and more generally, in LMICs) are inadequate for assessing the household economic burden of road traffic injuries. This is because they lack an appropriate comparison group so that we cannot, for example, infer from existing studies how much extra a household may be spending on healthcare due to road traffic injuries, relative to similar households. The significance of a comparison group is magnified if we note that households can cope with an adverse health event in a variety of ways–forgoing care, lowering OOP health expenses for illness for members of the household other than the injured person, lowering expenditures on non-medical items, incurring debt or selling assets, or changing the number of hours worked–and these responses may depend on household socioeconomic position, demographic composition or even characteristics that are unobserved. To our knowledge there is only one other study that has sought to address these questions in the South Asian context using a comparison group: Mohanan (2013) compared a sample of 84 households with bus accident injury cases to a set of 336 comparison households with similarly assessed risk of bus accident exposure based on frequency of bus travel, age, sex, and place of residence in the neighbourhood of the city of Bangalore (India) using regression methods [23]. The study concluded that despite high OOP health expenditure (which was equivalent to two months of household income) traffic injury-affected households were able to avoid a decline in non-medical consumption, but at the cost of significant increases in indebtedness.

Our study assesses the economic consequence of road traffic injuries on households in South Asia, specifically in Bangladesh, India, Nepal, Pakistan and Sri Lanka, using nationally representative household survey data from the World Health Survey (WHS). As in Mohanan (2013), our study attempts to link plausibly exogenous road traffic injuries and household level economic outcomes, such as OOP health spending, employment of the injury-affected individual, and effect on non-medical consumption, using a comparison group. We postulate that households containing individuals with road-traffic injuries incur higher OOP health expenditure, especially hospitalization spending, compared to unaffected households; secondly, in the absence of formal financial protection, the affected households rely on informal borrowing and/or selling assets to finance their higher OOP health spending. Finally, we hypothesize that the affected households experience negative employment effects and lower non-medical consumption. The multi-country scope of our study allows us to make inferences about the economic impacts of road traffic injuries in a broader set of countries (in South Asia), noted as a major gap in the existing literature [13]. Our analysis relies on a novel method to match households with an injured member to a set of comparison households, referred to as “coarsened exact matching”, to help construct closer matches of treatments and controls than matches based the commonly used method of propensity scores [24].

## Materials and Methods

### Data Source

The WHS is a set of nationally representative household surveys conducted by the World Health Organization (WHO) that collected observational data on socioeconomic and demographic characteristics of individuals and households, healthcare financing and healthcare use in 70 low, middle and high income WHO member countries during 2002–2003. The WHS data also included information on health conditions from one adult member (randomly chosen using Kish Tables) in each household, aged 18 years or over. Sample households were selected based on a random, stratified sampling procedure. The sampling procedure is described in detail elsewhere [25]. The interviews were conducted in person following written consent from the respondent and WHO’s ethical approval for the survey at each study site. For this study, Monash University Human Research Ethics Committee granted exemption from further ethical review (reference no. CF12/1442-2012000778). Our study sample included WHS data from five countries in South Asia: Bangladesh (5,942 households), India (10,692 households), Nepal (8,822 households), Pakistan (6,502) and Sri Lanka (6,805 households), consisting of 38,763 households in total.

### Matching Methods: Coarsened Exact Matching

To assess the impact of road traffic injuries on household economic outcomes, our analysis matched a household where a respondent reported being involved in a road traffic injury (traffic injury-affected households) to households where the respondent did not report a road traffic injury.

Matching was required because the exposure to the risk of traffic crashes is likely to depend on characteristics of household and individual respondents, including age, gender, community characteristics (such as road quality and availability of sidewalks) and other (truly random) influences. A popular approach to arriving at a comparison group is via the use of propensity scores, or the ‘likelihood’ of a household respondent suffering a traffic injury (using pre-treatment covariates consisting of household, individual and community characteristics in a logit model with an indicator for traffic injury as the outcome) [26]. Households with a member with traffic injury are then matched to comparison households with a similar propensity score. However, matching based on propensity scores can lead households with very different characteristics to get matched to each other. If instead, households were matched *exactly* on each of their observed characteristics, the consequence can be a very large loss of observations when the number of matching covariates is large and some variables are continuous, sometimes referred to as the curse-of-dimensionality [27].

An alternative matching approach we adopted is coarsened exact matching (CEM) which matches households *exactly* after ‘coarsening’; that is, after creating categorizations for continuous pre-treatment variables or creating coarser categories from finer categories [24]. For example, instead of exact age (in years), a coarsening entails specifying age-categories to which a respondent belongs–such as 0–14 years, 15–29 years, etc. Unlike matching based on propensity scores, CEM does not entail checking for covariates balancing, as households are matched *exactly* on each (coarsened) covariate and not on the propensity scores. This approach retains larger sample size for matching than matching *exactly* on all household characteristics. Although sample attrition can still occur when the number of such characteristics (matching variables) is large, the problem is significantly less serious than in the case of *exact matching* because real life survey data are less “independent” than theoretical considerations might suggest [28]. We performed analysis using STATA, version 12.1.

### Measurement of Road Traffic Injuries (Treatment Variable)

A treatment household was defined as one where the survey respondent reported suffering from ‘a bodily injury in a road traffic accident’ in the 12 months preceding the survey (injury-affected household) [29]. This definition covered crashes in which the respondent was involved in, either as the occupant of a motor vehicle, or when riding a motorcycle or bicycle, or walking. Based on this definition and using WHS data, the estimated prevalence of road traffic injuries was 4.90% (280 cases) in Bangladesh, 3.70% (327 cases) in India, 2.02% in Nepal (164 cases), 1.62% (121 cases) in Pakistan and 1.21% (112 cases) in Sri Lanka. The WHS estimates for Pakistan are comparable to data from a nationally representative survey data in Pakistan of 1.50% [10]. Although, comparable national surveys are unavailable for other countries in the region, it is estimated that the incidence of ‘serious injuries/deaths’ from road traffic crashes in Bangladesh and India is roughly 0.15%-0.25% [12]. Given estimates in the South Asian region of the ratio of minor injuries to serious injuries/deaths of roughly 4.40 [12], the WHS estimates for injury incidence for Bangladesh and India appear to be higher compared to other studies.

### Variables Used for Coarsened Exact Matching

The choice of variables used for matching is driven by the need to ensure that a key assumption of matching exercises–that conditional on the covariates used for matching, the likelihood of being injured is independent of potential economic outcomes (that is, the outcomes if the person were not actually injured). Thus, we choose variables that are likely to directly influence both the chance of being injured as well as the economic outcomes of interest [30]. Rubin and Thomas (1996) suggest that when in doubt, additional variables may be included to lower the risk of exclusion bias [31]. At the same time, inclusion of additional variables leads to fewer matches (the curse-of-dimensionality), requiring a balancing act between loss of observations and risk of exclusion bias.

#### Characteristics of individual respondents

These include age (in completed years); an indicator for sex (1 if respondent was female, 0 otherwise); an indicator for marital status (1 if the respondent was currently married or cohabitating, 0 otherwise); and an indicator for education of the respondent (1 if completed secondary school or above, 0 otherwise). It can plausibly be argued that people of different ages behave quite differently on roads; the sex of a person is linked not just to economic opportunities in South Asia but also to his likelihood of leaving his home and getting injured; education is associated both with economic outcomes and knowledge about risks related to traffic injuries; and marital status may underpin concerns about safer driving and concern for others, and may simultaneously indicate social linkages that can affect health expenditures.

#### Characteristics of other household members

Socioeconomic and demographic characteristics were used for household members other than the respondent. These included, specifically, the proportion of females in the household, the proportion of currently married members including cohabitating couples in the household, the proportion of children under five years of age in the household, the proportion of adults (18–59 years) in the household, the proportion of elderly (60 years and above) in the household, and the proportion of members who had completed secondary education or above. Again one could plausibly argue that the relative share of women, child and elderly in the household could influence both the likelihood of a male member going out and seeking work (and getting injured) as well as funds left over for other purposes, such as non-medical spending and OOP healthcare payments. The arguments for including marital status and educational status of other household members are also justified on grounds outlined previously.

In addition, we included the age of the household head (in completed years); an indicator for sex of the household head (1 if male, 0 otherwise); an indicator for marital status of the household head (1 if currently married or cohabitating, 0 otherwise); and an indicator for education of the household head (1 if completed secondary school or above, 0 otherwise). The characteristics of the household head are used as an indicator of economic status (for instance, female-headed households tend to be poorer than average) in the literature and a large literature suggests that higher economic status is likely to be associated with risks of injuries and the amount of health spending incurred on household members.

#### Other household characteristics

These included household size; an indicator of rural-urban location (1 if location of household was urban, 0 otherwise); and multiple indicators for living conditions. These were an indicator for improved drinking water sources (1 if the household had access to piped water, protected tube well or bore hole, or protected dug well or protected spring, or rainwater into tank or cistern, or collected water from tanker-truck or vendor, 0 otherwise); and indicator for improved toilets (1 if the household had toilet facilities with flush or piped sewage system, or flush to septic tank, or poor flush latrine, 0 otherwise) following WHO definitions [32]; indicator for clean cooking fuel (1 if the household used gas or electricity for cooking, 0 otherwise); indicator for household heating (1 if the household used heating during cold weather, 0 otherwise); indicator for type of floor (1 if the floor of the household was hard floor such as tile, cement, brick or wood, 0 otherwise); and indicator for type of wall (1 if the wall of the household was cement, brick, stone or wood, 0 otherwise). The inclusion of multiple indicators of living conditions helps achieving more precision on household economic status.

#### Country dummies

These include dummies for Bangladesh, India, Nepal and Sri Lanka (1 if the household belongs to a country, 0 otherwise) to capture variations (in traffic regulation, licensing regimes, road and health infrastructure, and health financing) in each country that are relevant to the likelihood of being in a traffic crash and its economic consequences.

### Measurement of Economic Outcomes

#### OOP health spending

The WHS reported OOP health spending in the four weeks preceding the survey in two ways—one as a single estimate and another in itemized form [33]. Single item questions tend to generate significantly lower aggregate expenditures than the sum of multiple disaggregated questions [34, 35]. For this reason, and also because of our interest in individual components of OOP health spending, we used the sum of itemized reports of health spending divided by household size (that is, per household member). We also used the ratio of OOP health spending to total household spending (as percent) as an outcome indicator.

#### Spending on drugs

We used OOP spending on drugs per household member in the four weeks preceding the survey, and the ratio of OOP spending on drugs to total household spending (as percent) in the four weeks preceding the survey.

#### Spending on hospitalization

We used OOP hospital spending (per household member) in the four weeks preceding the survey, OOP hospital spending (per household member) in the 12 months preceding the survey, and the ratio of OOP hospital spending to total household spending (as percent) in the four weeks preceding the survey.

#### Reliance on borrowing or sale of assets to finance health expenditure

The WHS collected information on methods households used to finance OOP health spending in the 12 months preceding the survey [33]. An indicator taking the value 1 if any household reported borrowing from a family or friend or from outside the household, or reporting selling of household items to pay for healthcare, 0 otherwise, was used.

#### Measure of catastrophic OOP health spending

We constructed two measures of catastrophic OOP health spending: an indicator variable that took the value 1 whenever the ratio of household OOP health spending to total household expenditure exceeded 20%, 0 otherwise [36]; and an indicator variable that took the value 1 when the ratio of household OOP health spending to a measure of household’s ‘capacity to pay’ (total expenditure minus subsistence needs) exceeded 40%, 0 otherwise using methods reported in Xu *et al* (2003) [37].

#### Employment

Two indicators for employment status were used: (a) an indicator for whether the respondent was working (1 if the respondent was government employee, or non-government employee, or self-employed, or employer, 0 otherwise) and (b) an indicator for the main reason of not working for pay (1 if the respondent was not working due to illness, 0 otherwise) [29].

#### Non-medical consumption expenditure

The WHS data recorded household consumption spending in the four weeks preceding the survey in two ways: one as a single aggregate measure and another in itemized form such as food, housing, education, insurance premiums and all other goods [33]. We constructed a measure of non-medical consumption expenditure of households by summing up itemized expenditures, excluding medical spending.

All expenditure estimates are reported in international dollar (I$) based on the World Banks’s purchasing power parity in 2003.

### Comorbid Conditions

Because there was the risk that our estimates of the household economic effects of road traffic injuries could be confounded by comorbid conditions such as depression [38, 39], we estimated linear regression models (with all our economic outcomes being the outcome variables for the regression models), an indicator for traffic injury-affected households and an indicator for presence of a depression (both diagnostic and symptomatic depression for which information were available in the WHS dataset [29]) on a dataset consisting only of matched households based on CEM.

### Sub-group analyses

We compared economic outcomes of road traffic injuries for (a) urban households with rural households; (b) households where the head of the household had completed at least secondary school with households where the education of the head of household was below secondary school as a proxy of socioeconomic status (SES); and (c) households where the injured individual was a female with households where the injured person was a male. To assess group-specific differences in economic burden, we estimated linear probability models, using the following ordinary least square (OLS) regression on a dataset consisting only of matched households based on CEM.

Y=α+β*RTI+γ*U+θ*RTI*U+ϵ

Here, *Y* is an economic outcome variable; *RTI* is an indicator variable with 1 indicating a road traffic injury-affected household, 0 otherwise; *U* is an indicator variable with 1 indicating households located in urban areas, 0 otherwise [depending on the sub-group considered, *U* was replaced by *G* (gender of the traffic injured individual), or by *E* (education of head of household) in which the injured person lived]; RTI*U is a product (interaction) of the two previous indicators; *ϵ* is an error term; and *α*, *β*, *γ* and *θ* are parameters to be estimated. The coefficient of the product was used to assess sub-group differences.

### Robustness Checks

We undertook three types of robustness checks. In particular, we re-estimated our results using fewer matching covariates. First, we dropped six household characteristics related to members in the household of the injured respondent (such as the share of children and the share of elderly in the household). In another scenario, we dropped an additional four covariates (marital status of household head, type of household wall, fuel used by the household, heating source in cold weather). Second, we re-estimated our results after excluding Bangladesh, the country with the largest reported injuries from traffic crashes in our sample to ensure that the results were not driven by one large country sample. Finally, we assessed our results after excluding the 1% of the households with the highest level of OOP health spending (per household member).

## Results

Table 1 presents summary statistics for the matching variables for three sets of households: injury-affected households, matched comparison households based on CEM and the full set of (unmatched) households. It is apparent that the means for matched households are considerably closer than when comparing the variable means of injury-affected households and unmatched control households.

**Table 1:** Summary of matching variables by traffic injury-affected and control households, South Asia, 2003.

Matching Variables	Treated	Control-Matched	Control-Unmatched
***Country Dummy***			
Bangladesh (%)	42.79 (35.97, 49.82)	53.72 (49.70, 57.71)	14.82 (14.44, 15.19)
India (%)	28.37 (22.35, 35.01)	20.23 (17.13, 23.61)	26.74 (26.27, 27.21)
Nepal (%)	17.31 (12.43, 23.15)	18.12 (15.16, 21.39)	23.91 (23.46, 24.36)
Pakistan (%)	6.25 (3.37, 10.45)	4.21 (2.77, 6.10)	17.56 (17.16, 17.96)
Sri Lanka (%)	5.29 (2.67, 9.27)	3.72 (2.37, 5.53)	16.98 (16.58, 17.38)
***Traffic Injury-affected Individual***			
Age (mean)	38.09 (36.54, 39.63)	38.04 (37.26, 38.82)	38.54 (38.39, 38.70)
Sex: Female (%)	21.63 (16.24, 27.86)	19.42 (16.37, 22.76)	52.96 (52.44, 53.49)
Education level: Secondary school and above (%)	15.87 (11.18, 21.55)	8.09 (6.06, 10.53)	29.84 (29.36, 30.32)
Marital status: Currently married (%)	92.31 (87.81, 95.54)	97.23 (95.63, 98.39)	77.71 (77.26, 78.14)
***Other Non-injured Household Members***			
Children under five years of age (%)	13.44 (11.06, 15.83)	16.51 (14.95, 18.07)	11.06 (10.88, 11.23
Adult members (%)	39.62 (36.59, 42.66)	38.36 (36.69, 40.03)	44.36 (44.08, 44.63)
Elderly members (%)	5.26 (3.37, 7.15)	2.78 (1.96, 3.60)	9.70 (9.51, 9.90)
Sex: Female (%)	56.29 (53.20, 59.39)	58.19 (56.50, 59.88)	49.96 (49.71, 50.21)
Education level: Secondary school (%)	5.10 (3.05, 7.14)	2.87 (1.98, 3.77)	12.06 (11.83, 12.29)
Marital status: Currently married (%)	35.76 (32.95, 38.57)	35.40 (32.99, 36.00)	37.83 (37.58, 38.09)
***Characteristics of Household Head***			
Age (mean)	40.50 (39.01, 42.00)	39.49 (38.74, 40.25)	45.07 (44.92, 45.22)
Sex: male-headed household (%)	98.08 (95.15, 99.47)	98.38 (97.04, 99.22)	90.23 (89.91, 90.55)
Education level: Secondary school and above (%)	14.42 (9.95, 19.95)	7.61 (5.64, 9.99)	29.32 (28.83, 29.81)
Marital status: Currently married (%)	96.63 (93.19, 98.64)	98.71 (97.47, 99.44)	88.65 (88.31, 88.99)
***Characteristics of Household***			
Household size (mean)	5.19 (4.96, 5.42)	4.95 (4.84, 5.06)	5.92 (5.89, 5.94)
Location: Urban (%)	20.19 (14.96, 26.30)	9.87 (7.63, 12.50)	25.90 (25.44, 26.36)
Floor (cement, tile, brick, wood) (%)	21.15 (15.81, 27.34)	10.36 (8.07, 13.03)	42.90 (42.38, 43.43)
Wall (cement, brick, stone or wood) (%)	26.44 (20.58, 32.99)	13.75 (11.14, 16.72)	51.73 (51.20, 52.27)
Improved water source (%)	97.12 (93.83, 98.93)	98.71 (97.47, 99.44)	89.87 (89.55, 90.19)
Improved latrine (%)	20.19 (14.96, 26.30)	10.19 (7.92, 12.85)	41.90 (41.38, 42.43)
Clean cooking fuel (%)	14.42 (9.95, 19.95)	6.96 (5.08, 9.26)	21.62 (21.18, 22.05)
Household heating in cold (%)	11.54 (7.53, 16.68)	12.46 (9.96, 15.32)	25.23 (24.77, 25.70)
***Sample***	***208***	***618***	***34*,*713***

*Notes*: Unweighted household level estimates are based on raw data from the World Health Survey for 2003. The data presented refer to the households, which responded to the survey question on whether or not a household member experienced any road traffic injury. 95% confidence intervals are reported in parentheses of each mean/proportion.

We found that the matched injured cases were slightly older than the unmatched injured cases (38.09 years vs 37.24 years), with a higher proportion of males (78.37% vs 75.74%), and much less educated (15.87% vs 38.35%) (lower proportion of individuals with secondary education and higher). However, a greater proportion of matched cases were married (92.31% vs 67.91%) and the average household size for matched cases was lower than of unmatched cases (5.19 vs 6.18). There was also a greater share of rural households in matched than unmatched cases (79.81% vs 63.79%). Among the five countries, Sri Lanka (89.32%) and Pakistan (89.25%) saw the highest share of traffic injury cases dropped followed by India (81.85%), Nepal (77.64%), and Bangladesh (67.87%).

Table 2 reports estimates of the household economic impacts of road traffic injuries, with and without adjusting for depression, the latter being our preferred estimates. Traffic injury-affected households experienced significantly higher levels of OOP health spending per member (I$0.75, *p*<0.01), higher OOP spending on drugs per member (I$0.30, *p* = 0.03), higher OOP hospital spending per member (I$0.29, *p*<0.01) in the last four weeks preceding the survey, and higher OOP spending per member on hospital care (I$1.82, *p* = 0.07) in the last 12 months preceding the survey. OOP health spending was also higher as a proportion of total household spending—by 3.80% points for aggregate OOP health spending (*p*<0.01), by 1.90% (*p* = 0.05) points for drug spending, and by 1.24% (*p*<0.01) points for hospital spending in the last four weeks preceding the survey.

**Table 2:** Economic Impacts of Road Traffic Injuries on Households, South Asia, 2003: Average Treatment Effect on the Treated (ATT).

Economic outcome indicators	*Unadjusted*			*Adjusted for Depression*		
	Outcomes for Treatment Households	Outcomes for Control Households	ATT (2)	Outcomes for Treatment Households	Outcomes for Control Households	ATT (2)
Per person OOP health spending in last four weeks (I$)	1.79 (1.41, 2.16)	1.02 (0.81, 1.24)	0.76** (0.22)	1.77 (1.37, 2.16)	1.01 (0.78, 1.24)	0.75**(0.22)
Per person expenditure on medicine in last four weeks (I$)	1.03 (0.81, 1.26)	0.73 (0.60, 0.87)	0.30** (0.13)	1.03 (0.79, 1.27)	0.73 (0.60, 0.87)	0.30**(0.13)
Per person hospitalization expenses in last four weeks (I$)	0.31 (0.19, 0.43)	0.02 (-0.05, 0.09)	0.29** (0.07)	0.31 (0.18, 0.43)	0.02 (-0.05, 0.09)	0.29**(0.07)
Per person hospitalization expenses in last 12 months (I$)	3.25 (1.54, 4.96)	1.39 (0.39, 2.38)	1.86* (1.01)	3.15 (1.35, 4.95)	1.33 (0.29, 2.37)	1.82* (1.02)
Borrowing or selling assets to meet health expenditure in one year (%)	46.63 (39.94, 53.33)	39.61 (35.73, 43.50)	7.02* (3.95)	44.60 (37.59, 51.62)	38.46 (34.40, 42.53)	6.14 (3.97)
Ratio of OOP health spending and total household expenditure (%)	14.11 (12.14, 16.08)	10.18 (9.04, 11.33)	3.93** (1.16)	13.80 (11.73, 15.87)	10.00 (8.81, 11.20)	3.80**(1.17)
Ratio of OOP medicine and total household expenditure (%)	9.85 (8.23, 11.46)	7.93 (7.00, 8.87)	1.92** (0.94)	9.82 (81.26, 11.51)	7.92 (6.94, 8.90)	1.90**(0.96)
Ratio of OOP hospitalization and total household expenditure (%)	1.33 (0.87, 1.79)	0.08 (-0.18, 00.35)	1.25** (0.27)	1.32 (0.84, 1.80)	0.08 (-0.20, 0.36)	1.24** (0.27)
OOP health spending share of total household expenditure at 20% cut-off	25.48 (20.05, 30.91)	18.15 (15.00, 21.30)	7.33** (3.20)	23.46 (17.77, 29.14)	17.01 (13.72, 20.30)	6.45**(3.21)
OOP health spending share of ‘capacity to pay’ at 40% cut-off	34.62 (28.46, 40.77)	26.86 (23.29, 30.44)	7.75** (3.62)	33.81 (27.35, 40.27)	26.41 (22.67, 30.15)	7.40**(3.65)
Employment of traffic injury-affected respondent (%)	78.37 (72.86, 83.87)	79.84 (76.64, 83.03)	-1.47 (3.24)	78.41 (72.63, 84.18)	79.86 (76.51, 83.20)	-1.45 (3.27)
Unemployment of traffic injury-affected respondent due to illness (%)	1.92 (0.37, 3.48)	1.11 (0.21, 2.01)	0.81 (0.91)	1.32 (-0.30,2.95)	0.77 (-0.17, 1.71)	0.55 (0.92)
Per person non-medical consumption expenditure in last four weeks (I$)	7.90 (4.34, 11.47)	8.77 (6.70, 10.84)	-0.86 (2.10)	8,01 (4.27, 11.75)	8.83 (6.66, 10.99)	-0.82 (2.12)
***Treatment (Control)***	***208 (618)***	***208 (618)***	***208 (618)***	***208 (618)***	***208 (618)***	***208 (618)***

*Notes*: Estimates are based on authors’ calculations using World Health Survey data. The data presented refer to the households, which responded to the survey question on whether or not a household member experienced road traffic injuries. Average treatment effects on household economic outcomes are estimated following the coarsened exact matching. For average treatment effect, standard error is shown in parenthesis with identification of statistical significance at the level of 5%** and 10%*. For treatment households and matched control households average treatment effects are shown with 95% confidence intervals in parenthesis. All expenditure estimates are in international dollars based on the World Banks’s purchasing power parity in 2003.

Measures of catastrophic OOP health spending were also higher in the injury-affected households: 6.45% (*p*<0.01) for the threshold of OOP health spending to total household spending ratio of 20%, and 7.40% (*p*<0.01) for the threshold of OOP health spending to household ‘capacity to pay’ ratio of 40%.

Our analysis did not suggest any differences between the road traffic injury-affected and control households in non-medical consumption expenditure (I$ = -0.82, *p* = 0.70) in the four weeks preceding the survey, nor did we find any statistically significant difference in measures of employment (-1.45% points, *p* = 0.66), or not working due to illness (0.55% points, *p* = 0.55)). Finally, although the proportion of households reporting borrowing or selling assets is higher in road traffic injury-affected households by 7.02% points (*p* = 0.08), the difference became smaller and statistically indistinguishable from zero once depression was adjusted for (6.14% points, *p* = 0.12).

Table 3 reports findings from our sub-group analyses. The columns in each table report the economic impact of traffic injuries belonging to specific sub-groups (*p*-values for the differences between comparison sub-groups are not reported in Table 3). We find that high SES households (where the head of household exceeded secondary schooling) incurred higher OOP spending on healthcare overall and on hospital care than low SES households (head of household without secondary schooling). Differences between the two groups were statistically indistinguishable from zero for all other outcomes at the 10% level of significance. In the comparison between rural and urban households, outcomes between the two groups were not significantly different except for catastrophic spending which was higher in rural areas. But per person expenditures on hospitalization in last four weeks were higher in urban households with the difference being statistically significant at 5% level. The data in Table 3 though, leans towards the finding that road traffic injuries’ impact on rural households to a greater extent than urban households for almost all indicators of OOP health spending. Our results also suggest gender differences in per person OOP health spending and per person expenditures on hospitalization in the four weeks preceding the survey with the difference being statistically significant at 5% level. Although differences in the other economic outcome indicators were statistically indistinguishable from zero, the results generally suggest that traffic injuries impact females to a greater extent than their male counterparts.

**Table 3:** Economic Impacts of Road Traffic Injuries on Households, South Asia, 2003: Sub-Group Analysis.

Economic outcome indicators	*Socioeconomic Status*		*Location*		*Gender*	
	Low SES	High SES	Rural	Urban	Male	Female
Per person OOP health spending in last four weeks (I$)	0.60** (0.24)	1.61** (0.58)	0.77** (0.25)	0.67 (0.50)	0.65** (0.25)	1.12** (0.48)
Per person expenditure on medicine in last four weeks (I$)	0.29** (0.14)	0.36 (0.35)	0.37** (0.15)	<-0.01 (0.30)	0.23 (0.15)	0.52* (0.29)
Per person hospitalization expenses in last four weeks (I$)	0.24** (0.08)	0.59** (0.18)	0.27** (0.08)	0.35** (0.16)	0.24** (0.08)	0.46** (0.15)
Per person hospitalization expenses in last 12 months (I$)	1.57 (1.10)	3.33 (2.67)	2.10* (1.13)	0.72 (2.27)	1.90* (1.15)	1.51 (2.17)
Borrowing or selling assets to meet health expenditure in one year (%)	6.37 (4.17)	5.67 (10.17)	6.17 (4.29)	6.86 (8.60)	5.77 (4.49)	7.39 (8.48)
Ratio of OOP health spending and total household expenditure (%)	3.61** (1.26)	5.03* (3.06)	4.56** (1.30)	0.75 (2.60)	3.30** (1.32)	5.58** (2.50)
Ratio of OOP medicine and total household expenditure (%)	2.13** (1.02)	0.70 (2.48)	2.63** (1.06)	-0.98 (2.12)	1.56 (1.08)	3.15 (2.04)
Ratio of OOP hospitalization and total household expenditure (%)	1.20** (0.29)	1.49** (0.72)	1.34** (0.30)	0.87 (0.61)	0.99** (0.31)	2.15** (0.58)
OOP health spending share of total household expenditure at 20% cut-off (%)	7.02** (3.46)	3.24 (8.43)	8.76** (3.57)	-2.75 (7.16)	4.79 (3.64)	12.45* (6.86)
OOP health spending share of household’s ‘capacity to pay’ at 40% cut-off (%)	7.50* (3.86)	7.55 (9.42)	10.63** (4.00)	-5.19 (8.01)	7.05* (4.13)	8.74 (7.79)
Employment of traffic injury-affected respondent (%)	-2.43 (3.52)	4.54 (8.58)	-0.84 (3.64)	-3.95 (7.30)	-1.44 (2.99)	-7.08 (5.64)
Unemployment of traffic injury-affected respondent due to illness (%)	0.74 (0.99)	-0.53 (2.41)	0.86 (1.02)	-0.68 (2.05)	0.09 (1.04)	2.21 (1.96)
Per person non-medical consumption expenditure in last four weeks (I$)	-0.32 (2.22)	-4.35 (5.41)	-0.25 (2.31)	-3.51 (4.64)	-0.94 (2.40)	-0.41 (4.52)
***Treatment (Control)***	***208 (618)***	***208 (618)***	***208 (618)***	***208 (618)***	***208 (618)***	***208 (618)***

*Notes*: Estimates are based on authors’ calculations using World Health Survey data. The data presented refer to the households, which responded to the survey question on whether or not a household member experienced road traffic injuries. Average treatment effects on household economic outcomes are estimated following coarsened exact matching. For each coefficient of average treatment effect, standard error is shown in parenthesis with identification of statistical significance at the level of 5%** and 10%*. All expenditure estimates are in international dollars based on the World Banks’s purchasing power parity in 2003.

Results from re-estimation after we drop some of the covariates used for matching are described in Table 4 and show that our conclusions are unchanged even with this adjustment. CEM analyses based on excluding the 1% of the households with the highest levels of OOP health spending (per household member) and Bangladesh also leave our main conclusions unchanged.

**Table 4:** Economic Impacts of Road Traffic Injuries on Households, South Asia, 2003: Average Treatment Effect on the Treated (ATT)-Robustness Checks.

Economic outcome indicators	(2) ATT (for 27 covariates)	(2) ATT (for 21 covariates)	(3) ATT (for 17 covariates)
Per person OOP health spending in last four weeks (I$)	0.76** (0.22)	0.86** (0.28)	0.52** (0.22)
Per person expenditure on medicine in last four weeks (I$)	0.30** (0.13)	0.25** (0.12)	0.14 (0.11)
Per person hospitalization expenses in last four weeks (I$)	0.29** (0.07)	0.43** (0.11)	0.26** (0.09)
Per person hospitalization expenses in last 12 months (I$)	1.86* (1.01)	2.21** (0.81)	1.50* (0.81)
Borrowing or selling assets to meet health expenditure in one year (%)	7.02* (3.95)	9.27** (2.90)	7.52** (2.36)
Ratio of OOP health spending and total household expenditure (%)	3.93** (1.16)	3.65** (0.88)	3.15** (0.75)
Ratio of OOP medicine and total household expenditure (%)	1.92** (0.94)	1.03 (0.69)	1.19** (0.56)
Ratio of OOP hospitalization and total household expenditure (%)	1.25** (0.27)	1.15** (0.26)	0.78** (0.25)
OOP health spending share of total household expenditure at 20% cut-off	7.33** (3.20)	9.35** (2.30)	8.71** (1.91)
OOP health spending share of ‘capacity to pay’ at 40% cut-off	7.75** (3.62)	7.36** (2.62)	05.50** (2.13)
Employment of traffic injury-affected respondent (%)	-1.47 (3.24)	1.35 (2.60)	1.84 (2.17)
Unemployment of traffic injury-affected respondent due to illness (%)	0.81 (0.91)	1.00* (0.57)	0.79 (0.53)
Per person non-medical consumption expenditure in last four weeks (I$)	-0.86 (2.10)	0.66 (1.37)	0.85 (0.81)
***Treatment (Control)***	***208 (618)***	***346 (1455)***	***490 (2570)***

*Notes*: Estimates are based on authors’ calculations using World Health Survey data. The data presented refer to the households, which responded to the survey question on whether or not a household member experienced road traffic injuries. Average treatment effects on household economic outcomes are estimated following the coarsened exact matching. For average treatment effect, standard error is shown in parenthesis with identification of statistical significance at the level of 5%** and 10%*. All expenditure estimates are in international dollars based on the World Banks’s purchasing power parity in 2003. Column (2) used five country dummies; characteristics of injured household member: age, indicator of female, secondary school completed and currently married; characteristics of other non-injured household members: indicator of under 5 years children, adults, elderly, female, secondary school completed and currently married; characteristics of household head: age, indicator of male, secondary school completed and currently married; other household characteristics: household size, indicator of urban location, floor, wall, improved water source, improved latrine, clean cooking fuel and household heating in cold. Column (2) used all covariates except characteristics of other non-injured household members; and Column (3) used all covariates except characteristics of other non-injured household members, indicator of currently married household head, and indicator of wall, clean cooking fuel and household heating in cold.

## Discussion

Our analysis suggests that road traffic injury-affected households in South Asia face a greater economic burden than a comparison group of similar households, based on CEM, where the respondent in the comparison household did not report a road traffic injury. This burden is primarily through incurring higher OOP health spending associated with hospitalization and drugs. However, the effect size is much smaller in our study relative to previous studies [12, 23]. Our analysis also shows that road traffic injury-affected households in South Asia experience OOP health expenditures that exceed commonly used catastrophic thresholds in the literature [37, 40].

Our results do not support the hypothesis that road traffic injury-affected households reduce their non-medical consumption in the face of economic shocks introduced by road traffic injuries. This conclusion differs from that of Aeron-Thomas *et al*. (2004) but is similar to Mohanan (2013). We did not find large effects of road traffic injuries on our indicators of employment, specifically whether the injured individual was currently working, or alternately, was not working due to illness. In this, our findings are similar to those of Mohanan (2013), but different from other studies showing that road traffic injuries are associated with reduced work-days and earnings [12, 14, 21–23, 41]. Our analysis suggests that increases in labour supply of the respondent are unlikely to explain the maintenance of non-medical consumption, with the caveat that we do not have data on hours worked, or on the labour supply of other household members to investigate this subject more deeply. It may be that our road traffic injury cases might not be severe enough to lead to significant decline in employment.

Because OOP expenses on healthcare are significantly increased in road traffic injury-affected households, the added expenses may partly have been financed from increased borrowing/debt. Our analysis suggests some reliance on borrowing or sale of assets to finance OOP healthcare expenditure by road traffic injury-affected households relative to controls [42]. Overall, nearly 46% of road traffic injury-affected households reported borrowing or selling assets to finance OOP health expenses in the year preceding the survey. However, between 39%-40% of comparison group households also reported borrowing and selling assets, so the estimated effects are typically small in magnitude, between 6%-7% points, and generally statistically indistinguishable from a null of no impact. In this, our results differ from Mohanan (2013) who found a difference of 32% points between road traffic injury-affected households and a comparison group, possibly because his analysis focused on more serious injuries (being based on individuals compensated by the government in bus accidents) [23]. Because the WHS has information only on whether households borrowed/sold assets to finance healthcare, not how much they borrowed (or sold assets for) our results may underestimate this category. It is also possible that non-medical expenses include transportation expenses for medical treatment, as the WHS specifically excluded these from responses on OOP health spending for medical care.

Sub-group differences in our analysis are not always statistically distinguishable from zero, possibly owing to the small sample sizes. Perhaps, the sharpest results are that high SES households spend more OOP expenditure on healthcare. Taken in their totality, however, our sub-group results suggest that low SES households, households in rural areas and households with female injury-affected respondents are more at risk of financial stress.

Our study has obvious limitations. Our analysis was based on self-reported household expenditure data which is subject to measurement error [43, 44]. The results of a test-retest study of the WHS specifically found that respondents in this survey tended to under-report total household expenditure, and over-report OOP health expenditure [35]. Moreover, information on many of the outcome variables, debt/borrowing/employment, was limited to yes/no responses, ruling out a more careful investigation of the impact of road traffic injuries. Because money is fungible, respondent self-reports of borrowing and selling assets for health expenses could potentially be biased. However, further analysis of WHS data show that the share of households reporting borrowing or asset sales for healthcare expenses is increasing in the share of OOP health expenses in total household spending: specifically, the mean OOP health expenditure share is 15% among those who borrow or sell assets versus 7% among those who do not. Thus, borrowing and assets sales do correlate well with household financial stress due to ill health. Concerns have also been raised about the commonly used measures of “catastrophic health spending” levels as these inadequately account for the fact that OOP health expenses reflect choices made by households (whether going for expensive private services or almost free government services). But these are commonly accepted and used measures that enable us to compare our findings with the broader literature. Our treatment variables were self-reported road traffic injury cases among household respondents and this may lead some road traffic injury cases to end up in control households. If so, our estimates of economic effects would be downwardly biased.

A final limitation is that a causal interpretation of matching methods such as CEM requires the conditional independence assumption (CIA)–that is, conditional on the matching covariates, the outcomes for households would be independent of the likelihood of road traffic injuries [45]. Despite our using a number of observable characteristics for matching, there may still be unobserved variables that could both influence selection into injuries and also influence outcomes. This remains a drawback, and moreover, the CIA assumption is not directly testable. By controlling exactly for a range of key observed confounders, omission of which would likely lead to a violation of CIA, our approach offers a useful practical method to capture the economic dimensions of road traffic injuries.

## Conclusions

Our paper explores the economic burden of non-fatal road traffic injuries on households in the five major countries of South Asia. Our conclusions point to a significant economic burden of road traffic injuries on households in South Asia, largely due to OOP spending on healthcare services but also highlights that the inclusion of a comparison group lowers estimates of the household economic burden of road traffic injuries. Road traffic injuries also lead to a significant increase in the proportion of households reporting catastrophic OOP health spending. Our analysis points to the need for financial risk protection for households affected by road traffic injuries in South Asia. Moreover, with the economic burden expected to rise in the future, prevention measures are warranted.
